# Organoid-Guided Synthetic Micropeptide Therapeutics to Target Ovarian Cancer Stem Cells

**DOI:** 10.3390/cancers18203289

**Published:** 2026-10-12

**Authors:** Bhavana Balayoganandh, Naisarg Gamit, Harshit Kallepalli, Bhawna Dev, Arun Dharmarajan, Sudha Warrier

**Affiliations:** 1 Department of Biotechnology, Faculty of Biomedical Sciences and Technology, Sri Ramachandra Institute of Higher Education and Research, Chennai 600116, Tamil Nadu, India; 2 Division of Cancer Stem Cells and Cardiovascular Regeneration, Manipal Institute of Regenerative Medicine, Manipal Academy of Higher Education (MAHE), Bangalore 560063, Karnataka, India; 3 Department of Radiology, Sri Ramachandra Institute of Higher Education and Research, Chennai 600116, Tamil Nadu, India; 4 Trichy SRM Medical College Hospital and Research Center, Trichy 621105, Tamil Nadu, India; 5 School of Human Sciences, Faculty of Life and Physical Sciences, The University of Western Australia, Perth, WA 6009, Australia; 6 Faculty of Health Sciences, Curtin Medical School, Curtin University, Perth, WA 6102, Australia; 7 Faculty of Life Sciences, Atria University, Bangalore 560024, Karnataka, India

**Keywords:** reproductive health, ovarian cancer, ovarian cancer stem cells, chemoresistance, patient-derived organoids, 3D disease modelling, peptide engineering, synthetic micropeptides, nanomedicine, targeted drug delivery, precision oncology

## Abstract

Ovarian cancer remains the most lethal gynecological malignancy, largely due to high recurrence rates and the development of therapy-resistant disease. Cancer stem cells (CSCs) contribute to treatment failure by promoting chemoresistance through signaling pathways such as Wnt/β-catenin, Hedgehog, and Notch, as well as through dynamic interactions with the tumor microenvironment (TME). Ovarian CSC (OCSC)-associated biomarkers, including CD44, CD24, CD133, and ALDH, have expanded opportunities for specific tumor characterization and biomarker identification. Effective targeting of OCSCs requires experimental models that preserve cellular plasticity and hierarchical organization. Development of synthetic matrices and chemically defined media has improved the reproducibility and scalability of three-dimensional (3D) organoid cultures for preclinical drug testing. CSC-derived organoids, established by enriching cells positive for CSC markers directly from patient tumor material, provide a 3D platform for studying OCSC-associated phenotypes and therapeutic responses. This review examines current understanding of ovarian CSC biology and the development of OCSC-derived 3D organoid models that can recapitulate cellular architecture and patient-specific drug responses. These 3D organoid models provide a guided platform for evaluating emerging therapeutics, such as micropeptides encoded by short open reading frames or engineered from functional tumor-suppressor protein domains selected to target OCSCs. Their small size enables modulation of intracellular signaling and highly specific protein–protein interactions. We further review micropeptide stabilization strategies and tumor-targeted delivery systems designed to enhance bioavailability. This review highlights how integrating patient-specific OCSC-derived organoid models with engineered micropeptides may accelerate the development of personalized treatment strategies for ovarian cancer while improving the preclinical evaluation of novel therapies.

## Simple Summary

Ovarian cancer mortality is strongly associated with the persistence of cancer stem cells, which promote chemoresistance through key signaling pathways. Targeting these cells requires experimental models that can support the study of CSC biology and therapeutic responses in a three-dimensional setting. This review discusses CSC biomarkers, the potential development of CSC-focused organoid models to assess treatment response, and emerging micropeptide-based therapies targeting CSC-associated signaling pathways. By integrating CSC biology, organoid technology, micropeptide design, and targeted delivery strategies, this review guides the development of more tailored approaches to ovarian cancer.

## 1. Introduction

Ovarian cancer (OC) accounts for around 4.4% of the overall cancer-related deaths in women and is the seventh most prevalent malignancy. OC-related mortality grew by 84.2% between 1990 and 2017 and is expected to grow further by 2040 [1]. A 2% annual decline in global OC incidence and mortality rates is required to reduce the burden by approximately 30% by 2040. Projections indicate that low-Human Development Index (HDI) countries may experience 100% increase, and very high-HDI countries may experience a 20–30% increase in cases and mortality. These projections show variation in OC burden across countries with different HDI levels, which reflects the need and access to precision oncology care [2]. Delayed symptom onset and the absence of reliable screening result in more than 70% of patients being diagnosed at an advanced stage, making OC the most lethal gynecological malignancy. Ovarian cancers are broadly classified into three major histological categories based on their cell of origin: epithelial tumors (~90%), stromal tumors (5–6%), and germ cell tumors (2–3%). Higher age, genetic susceptibility, infertility treatments, and family history are all established risk factors, whereas oral contraception, pregnancy, and lactation are protective factors, lowering the rate by one-third to two-fifths. Most cases occur between 50 and 69 years of age, with the highest crude incidence observed in individuals over 70 years [3]. Despite recent advances, such as treatment with PARP inhibitors, many patients still have poor survival rates, especially those with late-stage cancers [4]. Apart from cytoreductive treatment, clinical management of OC continues to face limitations due to a lack of tailored medicines for specific subtypes and drug alternatives for recurrence due to drug resistance. Cancer stem cells (CSCs) are a small subpopulation of tumor cells responsible for tumorigenesis, drug resistance, and post-treatment relapse. CSCs are thought to arise from tissue-resident stem cells or from differentiated tumor cells that undergo dedifferentiation and acquire stem-like properties, and various CSC-associated biomarkers have been connected to clinical outcomes and improving treatment [5,6]. Understanding CSC biology is central to addressing the shortcomings of conventional ovarian cancer management.

Two-dimensional (2D) cell cultures together with animal models have formed the experimental basis for investigating cancer biology and evaluating therapeutic responses. The simplicity of 2D cultures makes them valuable for mechanistic studies, but they poorly represent tumor heterogeneity and the multicellular interactions present within the tumor microenvironment (TME). Patient-derived organoids (PDOs) maintain key genetic and phenotypic features of their source tumors, providing a useful model for investigating tumor biology and therapeutic responses [7]. In parallel with these developments, short functional peptides encoded by small open reading frames (smORFs) [8] or derived from endogenous tumor suppressor proteins [9] have gained attention as potent regulators of cancer biology. Their small size enables them to selectively interfere with protein–protein interactions that control processes such as apoptosis, angiogenesis, and immune evasion. On the other hand, proteolytic breakdown, low stability, and poor bioavailability remain as major barriers. Delivery systems such as nanoparticle-based carriers, liposomes, hydrogels, and tumor-targeting formulations are being engineered to improve their stability and bioavailability within the tumor niche [10]. CSC-associated pathways represent potential targets for developing micropeptide-based therapeutics. CSC-derived organoid models generated by enriching cells positive for CSC markers offer a three-dimensional (3D) platform for evaluating therapeutic responses. Evaluating drug-delivery strategies in CSC-derived organoid models may further provide insights into pharmacodynamic responses and improve the translational value of these models. This review, therefore, brings together CSC biology for the marker- and function-based enrichment of CSCs from patient tumors, and organoid technology as a platform for micropeptide discovery, design, and delivery strategies to target ovarian cancer.

## 2. Mapping Cancer Stemness: Delving into the Chemoresistance of Ovarian Cancer

CSCs are a small but distinct group of cells within a tumor that exhibit stem cell-like features, including multipotency, the ability to remain in a quiescent, slow-cycling state, and self-renewal via asymmetric division. These cells are characterized by their ability to restore the original tumor’s heterogeneity during xenografting using as few as 100 cells in in vivo systems such as mice [11]. CSCs were first identified in the 1990s by John Dick and colleagues through studies using patient-derived acute myeloid leukemia (AML) samples transplanted into immunocompromised mice. This study demonstrated that a primitive CD34^+^CD38^−^ subset of cells can develop and propagate leukemia, whereas differentiated cells lack tumorigenic activity, indicating that leukemia arises from stem-like cells rather than the differentiated population and establishing cancer’s hierarchical organization, thereby laying the foundation for CSC biology [12]. Subsequent research expanded these findings in solid malignancies such as colorectal cancers (CRC), brain and breast cancers, widening their application beyond hematological tumors [11,13,14]. CSCs were also identified in OC patients in 2005, with CSCs that regenerated after treatment, reducing patient survival [15,16]. CSCs within tumors are described as a hierarchical organization, capable of tumor initiation and generating more differentiated tumor cell populations. The formation and maintenance of the CSC population are dynamic in process. Non-CSC tumor cells may acquire stem-like properties through dedifferentiation or phenotypic reprogramming in response to intrinsic and microenvironmental cues [5]. It is proposed that CSCs undergo asymmetric division during tumor initiation to maintain the stemness pool [17]. This process is controlled by the asymmetric segregation of polarity proteins (e.g., the Par complex and aPKC) and fate determinants (e.g., Numb and Prospero), which is coordinated by the mitotic spindle’s apical–basal alignment. This ensures that one daughter cell retains stemness, while the other commits to differentiation and undergoes rapid symmetric divisions to expand the proliferative pool and form the tumor bulk [18]. CSCs also have a dynamic interaction with the TME, especially after treatment, as demonstrated by therapy-induced senescence (TIS), which is tumor-suppressive but can later promote tumor progression. Senescent tumor cells release pro-tumorigenic senescence-associated secretory phenotype (SASP) proteins, which create an inflammatory milieu that promotes CSC survival, phenotypic plasticity, and relapse. As a result, using established CSC biomarkers for diagnosis and prognosis is crucial for identifying high-risk patients and therapy-induced resistance [5]. Recent evidence in OC has substantiated that cisplatin-induced senescence in high-grade serous ovarian cancer (HGSOC) cells generates SASPs that promote tumor cell detachment and metastatic dissemination through paracrine signaling, stressing the pro-tumorigenic effects due to chemotherapy [19]. In ovarian cancer, CSCs are identified by specific surface and functional markers, including ALDH1, CD24, CD44, CD117, and CD133, which have both prognostic and diagnostic value [20].

CD44 is a transmembrane hyaluronan receptor that has been widely investigated as a marker of Ovarian cancer stem cells (OCSCs) and other solid tumors. CD44 binds to hyaluronan and activates key signaling pathways such as PI3K/AKT and MAPK/ERK, promoting cell survival, motility, and stemness [21,22]. The prognostic value of CD44 expression in ovarian cancer is complex. High CD44 expression is frequently linked to carboplatin resistance and reduced survival [23], but its isoforms have distinct biological and prognostic roles. Higher CD44v6 expression is related to higher metastatic potential and worse overall survival [24,25] but CD44v8-10 correlates with better prognosis, while enzymatically cleaved variants are associated with aggressive disease and a poor prognosis [26]. Combined expressions of CD44 and CD117 as originally reported by Zhang et al., effectively distinguish epithelial OCSCs, highlighting CD44 as one of the targets of OCSC-associated phenotypes [27].

Another prominent biomarker of OCSCs, CD24, is a heavily glycosylated glycosylphosphatidylinositol (GPI)-anchored surface protein that was first identified in pre-B lymphocytes but is also expressed across multiple cell types. CD24 is overexpressed in numerous malignant tumor cells, particularly in ovarian carcinoma and CSCs, where it acts as a strong “don’t eat me” signal by engaging the inhibitory receptor Siglec-10 on macrophages, thereby shielding from phagocytic clearance and enabling immune escape. Thus, CD24 serves as a surface biomarker of the OCSC-associated phenotype and may indicate immune-evasive tumor characteristics by suppressing macrophage-mediated phagocytosis [28,29]. The CD24-Siglec-10 axis contributes to tumor-immune evasion, as Siglec-10 signaling inhibits T-cell receptor (TCR) activation and the phosphorylation of TCR-associated kinases such as Lck and ZAP-70. CD24 represents a link between OCSC-associated phenotypes and tumor immune regulation that can be simultaneously modulated [30]. CD24^+^ cells exhibit CSC-associated features by upregulating stemness-related genes and activating the JAK2-STAT3 signaling axis in-vivo [31]. CD24 activates PI3K/Akt, MAPK/ERK, and NF-κB pathways, leading to epithelial–mesenchymal transition (EMT) and cisplatin resistance in patient cohorts and cell-line models, enhancing survival, invasiveness, and chemoresistance. Furthermore, given that peritoneal dissemination represents the primary mode of progression in ovarian carcinoma, CD24^+^ cells promote intra-abdominal tumor dissemination and ascites formation in-vivo in OC, leading to faster disease progression and poor clinical outcomes [32]. CD24 is a critical upstream regulator of the Wnt/β-catenin pathway, interacting with β-catenin to enhance nuclear translocation and T-cell factor (TCF)/lymphoid enhancer factor (LEF) -dependent transcription of oncogenic target genes. In-vivo evidence shows that Apc ^Min/+^ and CD24^Min^^/−^ double knockout mice have dramatically reduced intestinal polyp growth, supporting the functional importance of this axis in colorectal cancer [33]. Based on the findings from our lab [34], the interaction between CD24 and β-catenin represents a critical oncogenic mechanism in OCSCs. Treatment with novel micropeptides SC301 and SC401, functional domains of the Wnt antagonist, secreted frizzled-related protein 4 (SFRP4), disrupted this CD24/β-catenin interaction. This disruption led to the inhibition of downstream Wnt target genes, suppression of EMT, reduction in autophagic flux, and restoration of chemosensitivity. Notably, SC-401 exhibited superior efficiency in targeting the CD24–β-catenin axis and reversing CSC-associated phenotypes in OC.

CD117 (c-Kit), a type III receptor tyrosine kinase (RTK), regulates essential cellular functions such as proliferation, survival, differentiation, and migration, and its overexpression has repeatedly been associated with poor clinical outcomes [27,35]. Chau et al. demonstrated that c-Kit is not only a CSC marker but also an active promoter of chemoresistance and tumorigenesis in OC. In-vitro and in-vivo studies showed that c-Kit suppression with siRNA or the tyrosine kinase inhibitor imatinib greatly reduced sphere formation, stemness marker expression, and tumor growth. c-Kit signaling enhances chemoresistance by activating the Wnt/β-catenin pathway and upregulating the drug efflux transporter ABCG2. Hypoxic conditions in the TME increase c-Kit expression through HIF-1α, promoting CSC survival [36].

CD133 (prominin-1), a pentaspan transmembrane glycoprotein, represents another well-documented CSC marker linked to OC growth [37,38]. Its expression is strongly regulated by epigenetic mechanisms, particularly DNA methylation, which helps to maintain CSC populations and increases tumor aggressiveness [39]. Ferrandina et al. demonstrated that ovarian cancers exhibit elevated levels of CD133-1 and CD133-2 epitopes, with CD133^+^ cells displaying higher clonogenic capacity as observed in primary cultures from OC tissues [40].

Aldehyde dehydrogenases (ALDHs), a family of detoxifying enzymes, protect cells by converting reactive and potentially toxic aldehydes to their corresponding carboxylic acids. Chemotherapeutic drugs like cisplatin and carboplatin work by causing DNA and protein damage, resulting in toxic aldehydes and reactive oxygen species (ROS) and increasing oxidative stress. Ideally, CSCs need to maintain low oxidative stress to protect their long-term genomic integrity. Thus, CSCs with high ALDH activity are better able to neutralize these cytotoxic byproducts and survive the chemotherapeutic pressure. Silva et al. reported ALDH activity in all ovarian cancers and cell lines, with ALDH^+^ cells demonstrating characteristic CSC features. Although CD133 expression was variable, the ALDH^+^CD133^+^ subpopulation was greatly enriched for tumor-initiating potential. As few as 1,000 CSCs isolated from patient tumors were sufficient to induce tumor formation in mice. The presence of ALDH^+^CD133^+^ cells in patient samples was substantially related to lower progression-free and overall survival, showing a strong relationship between this CSC fraction and aggressive disease [41,42,43]. The development of effective, personalized cancer therapies across ovarian and other malignancies is crucially dependent on the precise identification and stratification of CSC biomarkers, which is required for targeting the processes causing tumor survival and recurrence.

## 3. Limitations of Two-Dimensional Cultures and Animal Models in Ovarian Cancer Research

2D cell cultures grown as monolayers exhibit limited cell–cell and cell–matrix interactions, uniform exposure to media components, altered polarity that leads to aberrant cell behavior, and selection of phenotypes adapted to artificial in vitro conditions. As a result, they do not accurately recapitulate the in vivo microenvironment, wherein cells exist within a 3D architecture with dynamic spatial and biochemical heterogeneity [44]. On the other hand, traditional animal models are poorly suited for personalized cancer therapy because they fail to accurately replicate the human TME, immune context, and human vasculature. Furthermore, engraftment processes often alter tumor heterogeneity by creating a powerful evolutionary bottleneck, where only the most aggressive clones survive and proliferate [45,46,47]. Furthermore, time and throughput constraints, along with extremely high costs, make it impossible to test more than one or two drug combinations per model, which diminishes its practicality when treatment decisions are urgent. Last of all, the use of large numbers of animals for a single patient’s drug screening raises ethical concerns and is logistically unsustainable on a clinical scale. Due to these constraints, 3D cultivated organoids are more capable of simulating the physiological and pathological states of organ tissues, contain a wider range of cell types, and can produce functional “micro-organs.”

## 4. Three-Dimensional Organoid Systems for Translational Studies

H.V. Wilson (1907) originally illustrated the self-organizing capabilities of cells, wherein he demonstrated that cells from sponges could regenerate a complete organism, which served as the foundation for the concept of organoids [48]. Organoids are 3D structures derived from stem cells that self-organize into simplified, organ mimics. Adult stem cells (ASCs), induced pluripotent stem cells (iPSCs), and embryonic stem cells (ESCs) comprise some of the stem cell types that can generate these structures. Due to their emulative abilities of genetic characteristics, cellular attributes, and physiological behavior of the corresponding organs, organoids are helpful resources for drug screening, disease modeling, and developmental research [49]. PDOs generated from surgical specimens or malignant ascites provide a 3D platform that more closely reflects the native tumor architecture. Protocols have achieved success rates as high as 97% in establishing organoids from HGSOC, the most common and lethal subtype [50]. Combining organoid culture with organ-on-a-chip platforms and 3D bioprinting [46] represents a sophisticated ovarian cancer model. These systems could capture the biological complexity of ovarian tumors by incorporating immune and stromal components. Ovarian PDOs established directly from patient-derived tumors may contain CSC populations and provide a better disease model for conventional chemo-drug response. In contrast, ovarian CSC-derived organoids, enriched for CSC-specific cells, provide a platform for focused investigation of stemness-associated pathways. This review mainly formulates a framework for establishing and maintaining OCSC-derived organoids from patient tumors based on isolating cells with CSC phenotype. These models can be further developed in a modular manner by incorporating selected TME components such as stromal, vascular, or immune cells, depending on the biological question. Such approaches could enable controlled investigation of specific TME interactions while preserving the flexibility to study CSC biology or broader tumor responses.

## 5. Matrix Design and Culture Conditions for Cancer Stem Cells Derived Organoid Expansion

3D cell culture began to emerge in the early 1980s with the introduction of collagen- and laminin-rich extracellular matrices (ECM) as a step in recreating tissue architecture in-vitro [48]. Clevers et al. made a discovery in 2009, demonstrating that isolated crypts or Lgr5+ stem cells could form 3D intestinal organoids when embedded in Matrigel, a commercially available reconstituted basement membrane matrix derived from Engelbreth-Holm-Swarm (EHS) mouse sarcoma cells, together with growth factors [51,52]. This enabled the development of organoids from various tissues and organs, including the brain, liver, and kidney [49]. However, the batch-to-batch variability of Matrigel and its undefined composition may limit reproducibility and clinical translation. These limitations have driven the development of synthetic scaffold systems. For instance, polyethylene glycol (PEG)-based synthetic hydrogels can be customized with tissue-specific ECM cues and functionalized with adhesion peptides to support organoid development [53]. Hyaluronic acid (HA)-based gels offer tunable stiffness and biochemical properties, making them suitable for studying mechano-transduction and sensing in iPSC-derived bronchiolar and alveolar lung organoids [54], human spinal cord organoids, and blood vessel organoids [55]. Recent studies using decellularized tumor ECM and hydrogel demonstrate improved organoid fidelity in breast cancer, hepatocellular carcinoma (HCC), and cholangiocarcinoma (CCA), largely due to preservation of tumor-specific ECM cues that are lost in synthetic scaffolds [56,57,58].

Generating CSC-derived organoids requires a coordinated series of steps, starting with CSC isolation and followed by the selection of scaffold materials with tunable stiffness that mimic the local TME. Long-term cultivation of CSC-derived organoids requires specific growth factor cocktails that sustain CSCs and prevent non-stem lineages from outcompeting them. Selective supplementation of growth factors and TME components in CSC-derived organoids could help to maintain self-renewal and also introduce vascular or immune components, which would provide a more flexible and customized drug screening platform. Growth factor composition is a critical determinant influencing survival and self-renewal in cancer organoid cultures. Effective media formulations must support the retention of tumor characteristics while promoting proliferation [59] as summarized in Table 1.

Van de Wetering et al. (2015) established a living organoid biobank from CRC patients that retained key genetic and phenotypic characteristics of the original tumors and could be expanded and maintained for further drug-response experiments [67]. Their work further demonstrated the importance of Wnt signaling in organoid maintenance, as inhibition of Wnt secretion using the porcupine inhibitor IWP2 strongly affected an RNF43-mutant PDO. Although this study was conducted using conventional CRC-PDOs, these findings illustrate that general organoid culture principles may be applicable to maintaining CSC-derived organoids, including the use of Wnt3A and Wnt amplifiers such as R-spondin 1 [67].

Hence, formulation of CSC-derived organoid culture media can be guided by a set of core biological principles that collaboratively maintain stemness and enable expansion. Activation of Wnt signaling, mediated by Wnt3A, plays a central role in sustaining stemness and self-renewal [68]. Mitogenic support is further enhanced by tyrosine kinase receptor ligands such as EGF and bFGF, which promote proliferation [69], while BMP signaling can be inhibited using agents such as Noggin, a BMP antagonist, to prevent premature epithelial differentiation and support long-term maintenance [51,70,71]. ROCK inhibitors such as Y-27632 are commonly used in early culture phases to increase the survival of dissociated CSCs, which readily undergo anoikis [72]. Adequate metabolic and nutrient support, provided by supplements including insulin, B27, N2, and antioxidants, is required to minimize metabolic stress [59].

HGSOC is the most prevalent and aggressive OC, with significant intratumoral heterogeneity and a high risk of intraperitoneal dissemination. Although standard treatment with cytoreductive surgery followed by platinum-taxane chemotherapy produces favorable initial response incidences, more than 70% of patients eventually relapse with chemoresistance [73]. The cellular origin of HGSOC remains debated, with evidence suggesting that both the ovarian surface epithelium and the fallopian tube epithelium could be potential origins of tumor initiation. During this process, CSC-like populations are thought to emerge and expand within both primary tumor sites and metastatic niches, including spheroid structures in malignant ascites. Spatially diverse environments, ranging from hypoxic tumor cores to anchorage-independent ascitic aggregates, impose selection pressures on CSC maintenance. The distribution of CSCs is not uniform, but they are enriched in specific niches, including perivascular regions, hypoxic zones, and the invasive tumor front, where TME cues support their phenotype [74,75,76]. Experimental strategies focus on utilizing the characteristics of CSCs present within OC patient material, among which marker-based isolation via fluorescence-activated cell sorting (FACS) is mostly used. This technique enables high-throughput, quantitative isolation of live cells based on the presence or absence of specific stemness-associated epitopes. Commonly used markers in OCSCs include CD133 and CD44 in combination with CD117 and high enzymatic activity of ALDH1 (Aldehyde Dehydrogenase 1), a functional marker measured by the ALDEFLUOR assay [27,43,77]. An alternative functional assay to enrich OCSCs is the side population technique, which leverages the heightened ability of CSCs to efflux Hoechst 33342 dye via ATP-binding cassette transporters, resulting in a distinct population detectable by flow cytometry [78,79]. Given the presence of CD24 surface markers, where its expression is correlated with the survival and prognosis of a patient [32,80] exploiting the presence of CD24 to enrich for the OCSC population, especially by Magnetic-Activated Cell Sorting (MACS) for rapid enrichment or by FACS within the tumor bulk, is a robust method for generating CSC-derived organoids, providing a relevant model for studying this aggressive cell subset.

In contrast to marker-dependent isolation techniques like FACS, the cancer tissue-originated spheroid (CTOS) approach offers a non-selective strategy that prioritizes the preservation of native tissue architecture. In this technique, a gentle mechanical dissociation of patient-derived tissue is followed by size-based filtration and culture under non-adherent conditions. Cell aggregates formed from malignant tissue, which have strong cell–cell connections, are more likely to survive and expand as spheroids in these conditions, whereas stromal-derived aggregates often fail to grow over time. Serial passaging gradually enriches tumor-derived spheroids, thus the name “cancer tissue-originated spheroids” (CTOS). Unlike traditional isolation procedures, which target individual cells based on surface markers, the CTOS method selects intact multicellular structures, suggesting that the functional CSC unit is best kept within its natural architectural environment. CTOS is a specialized version of primary sphere culture that promotes the retention of native cell–cell interactions [81,82].

Ooft et al. demonstrated that chemotherapy responses observed in PDOs derived from metastatic CRC samples could predict the clinical response to conventional chemotherapeutic agents. The drug responses observed in PDOs were compared with the clinical responses of the corresponding patients receiving the respective treatment regimens. This showed predictive value for irinotecan-based treatment [83]. This approach provides a basis for assessing chemotherapeutic agents in combination with emerging therapeutics such as micropeptides using CSC-derived organoids for patient-specific therapeutic responses. The design and optimization of micropeptide-based therapeutics developed to target OCSCs is discussed in the subsequent sections.

## 6. Endogenous smORF-Derived Peptides and Chemically Synthesized Micropeptides as Emerging Modulators of Oncogenic Signaling

While advances have been made in understanding genetic drivers like TP53 and BRCA1/2 mutations, many aspects of cancer biology remain incompletely understood. RNA transcripts that are considered to be ‘non-coding’ such as long non-coding RNAs (lncRNAs), circular RNAs (circRNAs), and primary microRNAs (pri-miRNAs) were considered incapable of encoding proteins. This view has changed with the discovery of short peptides translated from small open reading frames (smORFs), referred to as small ORF-encoded peptides (SEPs). These are polypeptides typically 20-100 amino acids in length, translated from smORFs located on transcripts previously annotated as non-coding RNA. Screening techniques such as ribosome profiling (Ribo-seq) and mass spectrometry have expanded the catalog of previously unannotated bioactive peptides. These polypeptides are now recognized as critical regulators of cellular physiology, such as mitochondrial metabolism and calcium signaling [84,85]. A significant proportion is derived from smORFs within lncRNAs [86]. This review section discusses peptides encoded by smORFs as endogenous sources, whereas those derived from functional domains of larger proteins and chemically synthesized are referred to as “micropeptides.” Although these categories differ in their biological origin, both represent peptide-based modulators with potential relevance to cancer biology and therapy.

Table 2 provides a brief overview of the agonistic and antagonistic roles of peptides from endogenous sources across various cancers, based on cell line studies and clinical validation. Collectively, these examples demonstrate that peptides can exert either tumor-suppressive or oncogenic functions depending on their molecular context.

Cancer development is driven by genetic alterations that disrupt protein signaling networks through the inactivation of tumor suppressor genes (TSGs) and activation of oncogenes. Full-length proteins encoded by TSGs often contain functional domains that interact to mediate interactions with signaling partners involved in apoptosis, proliferation, angiogenesis, and immune surveillance. Understanding such protein domains has enabled the development of synthetic micropeptides as targeted therapeutics. These synthetic micropeptides are chemically designed to mimic the biological activity of specific domains within larger proteins, particularly tumor suppressor proteins [97].

Many strategies targeting stemness-associated properties, such as high ALDH levels, in OCSC populations have been investigated. ALDH inhibitors such as 673A and CM037 showed preferential suppression of CSC viability in OC. ALDH inhibition provides a strategy to target the enzymatic phenotype associated with OCSCs [98]. While naturally occurring smORF- or lncRNA-derived peptides represent as direct endogenous regulators, in this review, we discuss the strategic perspective of synthetically designing micropeptides to target specific vulnerabilities in oncogenic signaling. These micropeptides are derived from functional domains of larger proteins, particularly from TSGs, which function to inhibit oncogenic signaling, induce apoptosis, and modulate stemness pathways in OC. By retaining the active regions of their parent proteins, these micropeptides offer a targeted approach to restore lost tumor-suppressive activity. Sattler et al. developed peptides mimicking the BH3 domain of pro-apoptotic proteins to inhibit Bcl-2 family members [99], whereas Kanovsky et al. [100] designed peptides derived from the MDM2-binding domain of p53 that are independent of p53-induced cell death. These examples reflect the growing momentum in developing synthetic peptides as therapeutics in cancer biology, which are further discussed in Table 3.

## 7. Rational Design of Synthetic Micropeptides Targeting Oncogenic Pathways

Tumor-suppressor proteins frequently function through well-defined structural motifs, cysteine-rich domains, β-propellers, transmembrane regions, interaction interfaces, and short linear motifs (SLiMs). These motifs mediate protein–protein interactions (PPIs), receptor binding, signal inhibition, or scaffolding functions. Importantly, many anti-tumorigenic effects map to short, discrete regions, often 10–40 amino acids long [110]. Designing synthetic micropeptides that mimic these motifs allows selective reactivation of tumor-suppressive signaling in cancer cells, even when the full-length protein is absent or mutated [111]. This approach is conceptually similar to BH3 mimetics that emulate the pro-apoptotic BH3 domain of BCL-2 family proteins [112]. Strategically designed tumor suppressor peptides should aim to:

• Disrupt oncogenic PPIs (e.g., β-catenin interactions [34], MDM2–p53 binding [100]).
• Restore tumor-suppressive function lost during epigenetic silencing of TSGs [111].
• Block nuclear translocation of oncogenic transcription factors [113].
• Inhibit pro-survival pathways such as Wnt, PI3K/AKT, NF-κB, or Hippo [114,115].
• Induce apoptosis or loss of stemness in cancer stem cells [115].

One advantage of synthetic micropeptides is that they do not depend on endogenous gene expression. In tumors where promoter hypermethylation, gene mutations, or copy-number loss inactivate TSGs, exogenously delivered micropeptides can restore selected tumor-suppressive functions independently of transcription or translation.

## 8. Stabilization Strategies in Micropeptide Design

Micropeptides require chemical and structural stabilization to achieve effective antitumor activity. Within solid tumors and CSC-enriched microenvironments, where hypoxia, elevated protease activity, and dense ECM restrict peptide stability and tissue penetration, micropeptides must be both chemically stable and effectively transported. Addressing these challenges has shifted peptide development from simple linear sequences to structurally optimized molecules without compromising biological activity [116]. Table 4 summarizes the challenges affecting the pharmacokinetics, efficacy, and delivery of micropeptides, alongside current engineering solutions.

Stereochemical modification is the most widely used approach for improving micropeptide stability, notably through the substitution of D-amino acids. Endogenous proteolytic enzymes typically exhibit a stereospecific preference for levo-configured residues; consequently, replacing these residues with their D-enantiomers imparts a degree of resistance to protease-mediated cleavage. Partial D-substitution enables selective protection of cleavage-prone zones, while fully D-configured peptides can attain proteolytic resistance. When guided by computational models, D-configured peptides can maintain their target specificity, which makes them suitable for systemic application [117,128]. Cyclization represents peptide stability by restricting conformational flexibility. Head-to-tail cyclization protects terminal residues from exopeptidase action while maintaining a compact 3-D structure. Cyclized peptides frequently display membrane permeability and lower clearance rates, demonstrating better pharmacokinetics [130].

Beyond cyclization, peptidomimetic design provides an additional strategy for stabilizing bioactive motifs while overcoming the limitations of native peptide backbones. Adding β-amino acids, aza-peptides, and other non-canonical residues improves resistance to enzymatic degradation [122]. Stapled α-helices boost secondary structure with covalent cross-links, retaining essential side chain orientation for target engagement that allows micropeptides to retain domain-like functionality while being stable in biological environments [125].

**Figure 1:**
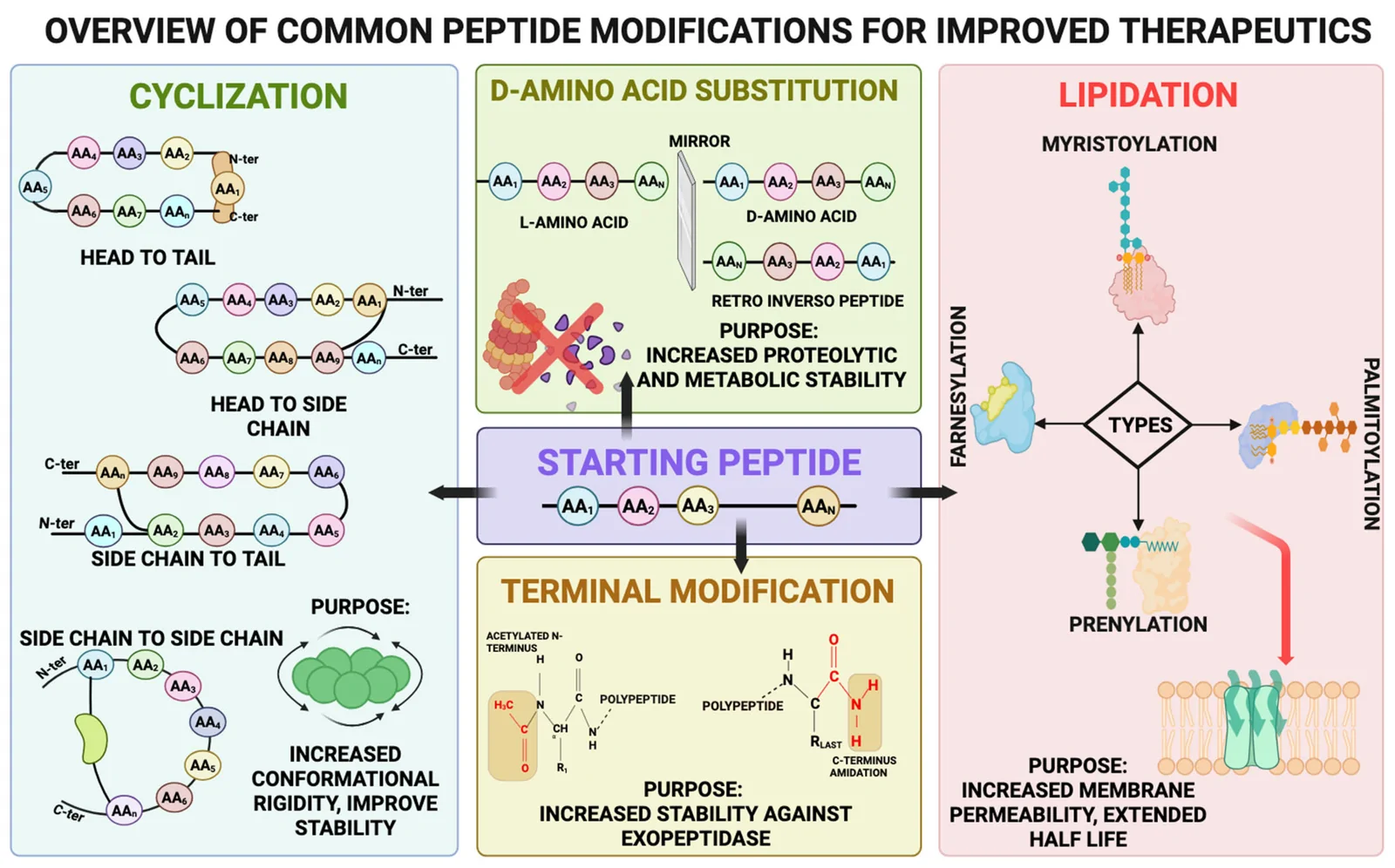
Major chemical modification strategies for improving the performance of micropeptides. Schematic overview of chemical modifications employed to optimize peptide-based therapeutics for improved pharmacokinetic and pharmacodynamic performance. (**Left panel**) Cyclization strategies, including head-to-tail, head-to-side chain, side chain-to-tail, and side chain-to-side chain cyclization, thereby increasing structural rigidity and resistance to proteolytic degradation. (**Upper middle panel**) D-amino acid substitution and retro-inverse peptide design replace naturally occurring L-amino acids with their D-enantiomers to maintain biological activity. (**Lower middle panel**) Terminal modifications, such as N-terminal acetylation and C-terminal amidation, protect peptides from exopeptidase-mediated degradation and prolong peptide half-life in-vivo. (**Right panel**) Lipidation strategies, including myristoylation, palmitoylation, prenylation, and farnesylation, improve membrane affinity and cellular uptake. These peptide engineering approaches enhance therapeutic efficacy, making them valuable strategies for next-gen peptide-based targeted cancer therapeutics. (Created in BioRender. Balayoganandh, B. (2026) https://BioRender.com/5q7683x; Accessed 28 July 2026).

Modification of peptide termini provides an additional means of improving stability. N-terminal acetylation or alkylation [127] and C-terminal amidation [118] protect peptides from aminopeptidase and carboxypeptidase activity, respectively. Terminal capping does not alter peptide structure dramatically but improves pharmacodynamics [119]. Lipidation further enhances peptide stability by improving membrane interactions and bioavailability. Conjugation of hydrophobic moieties, such as palmitoyl or stearoyl chains, as well as lipid-based modifications such as myristoylation and prenylation, improves peptide interactions with cellular membranes and serum proteins, thereby limiting renal clearance and enzyme breakdown. Lipidation has been successfully applied to anticancer peptides targeting intracellular signaling pathways [120]. Chemical modification is often complemented by delivery-based stabilization using encapsulation in nanocarriers, polymeric nanoparticles, and liposomes [122,127]; vault nanoparticles and exosome-mimetic vesicles [129,130]; or self-assembling hydrogels [119], which protect micropeptides against proteolysis and enable controlled release in in-vivo. Many nanocarriers are engineered to respond to tumor-associated stimuli such as acidic pH, redox gradients, or elevated protease activity, thereby promoting preferential release within the TME. Delivery systems frequently work in tandem with chemical stabilization, allowing peptides to maintain biological function. Such spatiotemporal control is particularly relevant for targeting CSC niches, which are often characterized by hypoxia and altered ECM composition. Delivery carriers can shield micropeptides from immune recognition while facilitating intracellular transport, thereby integrating stabilization, targeting, and cellular uptake within a single platform [131]. Figure 1 illustrates the structural optimization strategies commonly employed to improve the pharmacology of micropeptides. Rational modifications collectively enhance peptide stability, proteolytic resistance, and membrane permeability in vivo while maintaining biological activity.

Cell-penetrating peptides (CPPs) are complexes in which short peptides such as Trans-activator of Transcription (TAT) [132], penetratin, and poly-arginine sequences are conjugated to active micropeptides that engage transiently with membrane components, allowing for energy-independent or endocytotic uptake. CPPs facilitate functional intracellular delivery by reducing proteolytic degradation, accelerating membrane translocation, and reducing extracellular retention time [121]. Furthermore, CPP conjugation frequently increases peptide solubility without requiring extensive chemical modifications to the motifs. Certain CPPs also promote endosomal escape, thereby subverting degradation and ensuring the delivery of intact, bioactive micropeptides to cytosolic or nuclear targets, which is necessary for therapies targeting intracellular oncogenic signaling pathways [126]. When conjugated to micropeptides, these short sequences improve solubility, prevent aggregation, reduce cardiotoxic load, and enhance resistance to enzymatic degradation in the extracellular milieu [133,134]. CPPs can be implemented in a modular and customizable manner, for example, by using cleavable linkers to release the active peptide intracellularly, thereby reducing off-target effects and systemic toxicity [121]. Computational modeling approaches have become increasingly popular as a solution to overcome intrinsic limits of native peptides, such as short in-vivo half-life, low target specificity, and unwanted pro-tumorigenic activities. As comprehensively reviewed by D’Annessa et al. 2020, rational mimetic design incorporates non-natural amino acids, backbone modifications, cyclization, and conformational constraints to stabilize bioactive secondary structures involved in protein–protein interactions while improving stability and permeability [123]. Structure-guided computational approaches, such as peptide-protein docking combined with molecular dynamics (MD) simulations, are central to this process: docking generates and refines candidate binding structures, while MD simulations assess conformational stability through structural metrics such as hydrogen-bond persistence and root-mean-square deviation (RMSD). Binding free energies can subsequently be estimated using post-processing methods such as MM-GBSA or MM-PBSA, providing an approximate thermodynamic assessment of peptide-target interactions [123]. Although primarily discussed in the context of general peptides, these principles are immediately applicable to the rational design of micropeptides targeting oncogenic signaling networks, particularly CSC-associated pathways driven by abnormal protein–protein interactions [123]. Advances in proteogenomics and peptidomics have accelerated the discovery of regulatory micropeptides, especially when combined with computational protein design and high-throughput peptide libraries. Synthetic micropeptides engineered from functional domains of endogenous tumor suppressor proteins represent a targeted approach to restore specific tumor-suppressive signaling pathways disrupted during cancer progression.

## 9. Targeted Delivery Strategies for Micropeptide Therapeutics

Achieving efficient in-vivo delivery remains a major obstacle to the clinical development of micropeptides [135]. Due to their small size and vulnerability to proteolytic degradation, delivery platforms are necessary to ensure efficient accumulation at target tissue, particularly in therapy-resistant populations such as CSCs [136]. The following sections examine current strategies for improving targeting specificity and in vivo efficacy.

## 10. Nanocarriers and Formulations

Protecting therapeutic micropeptides from rapid degradation has driven the development of diverse nanocarrier-based delivery systems. Typically measuring 1–100 nm in diameter, nanocarriers improve drug delivery by enabling targeted delivery while reducing systemic toxicity. Nanocarriers can encapsulate a variety of therapeutic payloads, including hydrophobic and hydrophilic medicines, nucleic acids, antibodies, proteins, and peptides, protecting their pharmacokinetic properties [137,138]. The choice of nanocarrier system depends on its encapsulation efficiency, controlled release kinetics, and adaptability to the TME. Major nanocarrier platforms currently investigated for therapeutic micropeptide delivery are summarized below.

Among nanocarrier systems, liposomes are widely used because their phospholipid bilayers efficiently encapsulate both hydrophilic and hydrophobic cargos. Their phospholipid composition resembles biological membranes, protecting them from enzymatic degradation. Peptide-loaded liposomal formulations reduce off-target exposure while increasing tumor-specific delivery in preclinical cancer models [139]. Surface modification with polyethylene glycol (PEGylation) optimizes liposomal function by lowering opsonization and clearance by the reticuloendothelial system, extending systemic circulation compared to conventional liposomes [140,141]. PEG-coated liposomes, often termed “stealth liposomes,” avoid rapid recognition and uptake by phagocytic cells of the mononuclear phagocyte system, resulting in extended blood half-lives [142]. Beyond extended circulation, liposomes can be functionalized with targeting ligands such as folic acid, peptides, or antibodies, allowing for active targeting of tumor cells and CSC populations. Ligand-decorated liposomes selectively activate receptors overexpressed on CSCs, such as CD44, EpCAM, and integrins, increasing intracellular delivery of encapsulated micropeptides [143,144,145]. Likewise, pH-sensitive and fusogenic liposomal formulations have been designed to facilitate endosomal escape, which is essential for micropeptides targeting intracellular signaling pathways such as the epidermal growth factor receptor (EGFR) [146]. Similarly, pH-responsive liposomes functionalized with VEGFR2-targeting peptides have demonstrated cytoplasmic drug delivery in naturally VEGFR2-expressing HUVECs compared with HEK293 cells and have further been shown to be effective for targeting tumors in HT-29 xenograft models [147]. These modifications demonstrate how liposomes can be used for specific target and micropeptide delivery

Polymeric nanoparticles, particularly PLGA-based systems (poly (lactic-co-glycolic acid)), provide structural stability together with controlled drug-release kinetics. Their ability to co-deliver chemotherapeutic compounds and small-molecule drugs facilitates synergistic targeting of CSCs and tumor-supportive stroma. Hyaluronic acid-coated nanoparticles containing paclitaxel and salinomycin, targeting CD44^+^ cells, successfully disrupted both tumor cells and CSCs. Likewise, co-delivery of docetaxel and salinomycin using PLGA/TPGS nanoparticles reduced stemness and anti-CSC activity in spheroid models [148,149]. This adaptability of polymeric nanoparticles makes them well-suited for combination, spatial control, and long-term drug exposure in the TME.

**Figure 2:**
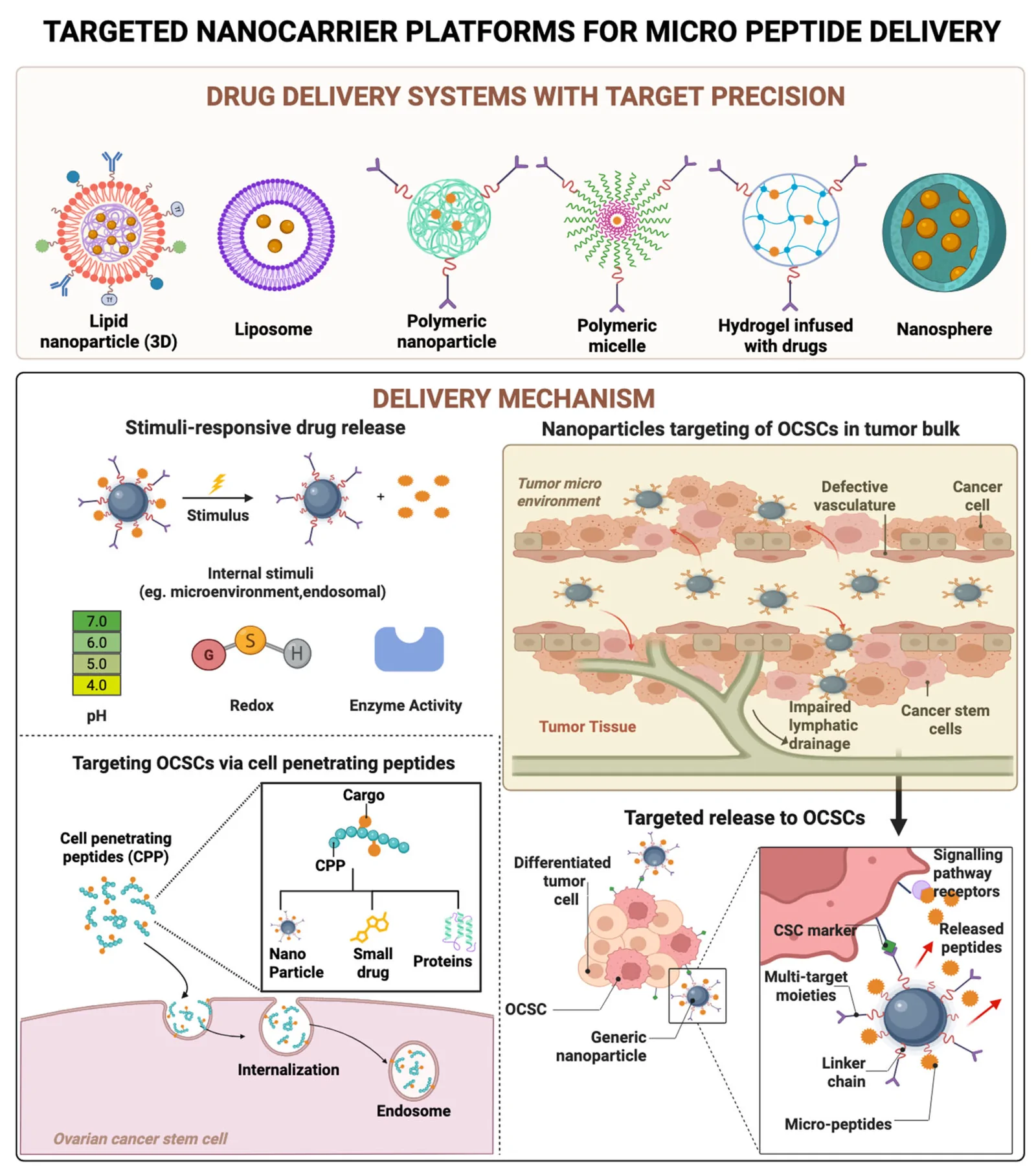
Advanced nanocarrier-based delivery strategies for targeted micropeptide therapeutics. Schematic representation of advanced nanocarrier platforms and their mechanisms for selective delivery of therapeutic agents to OCSCs. (**Top panel**) Overview of nanocarrier systems employed for targeted drug delivery, including lipid nanoparticles, liposomes, drug-loaded hydrogels, and nanospheres. These delivery platforms can be functionalized with targeting ligands to enhance specificity and increase intratumoral accumulation while minimizing off-target toxicity. (**Middle left)** Stimuli-responsive drug delivery systems enable controlled drug release in response to tumor-associated internal stimuli, including acidic pH, altered redox conditions, and enzyme activity, thereby improving site-specific drug release while minimizing systemic toxicity. (**Bottom left**) CPPs facilitate the intracellular delivery of nanoparticles and their therapeutic cargo by promoting membrane translocation and endocytic uptake into OCSCs. Following internalization, the released payload, which has nanoparticle-encapsulated micropeptides, small-molecule drugs, or whole proteins, can modulate CSC-associated signaling pathways. (**Bottom right**) Nanoparticles functionalized with CSC-specific targeting ligands selectively recognize OCSCs through interactions with cell surface markers. Following cellular uptake, the therapeutic payload, including micropeptides, is released via linker cleavage to modulate CSC-associated signaling pathways (Created in BioRender. Balayoganandh, B. (2026) https://BioRender.com/behso57; Accessed 28 July 2026).

Hydrogels provide a biocompatible platform for sustained and localized delivery of therapeutic peptides. Injectable polymer-nanoparticle hydrogels like cyclodextrin nanoparticles offer sustained release, high biocompatibility, and low off-target toxicity, allowing doses to remain at the disease site [150]. For cancer applications, hybrid hydrogels encapsulating paclitaxel delivery achieved sustained release and cytotoxicity in OC, significantly surpassing non-targeted or free-drug controls [151]. More importantly, TME-responsive hydrogels with periodic drug release have been found to regulate epithelial–mesenchymal transition (EMT) and angiogenesis [152]. Figure 2 illustrates an overview of advanced nanocarrier-based strategies for targeted micropeptide delivery. Together, these nanocarrier developments have complementary benefits that can be tuned to promote targeting precision in ovarian cancer. Integration with tumor organoid systems enables translational evaluation of micropeptide bioactivity, which aids in the development of nanocarrier design for enhanced penetration, controlled release, and target specificity.

## 11. Future Perspectives

Ovarian cancer develops within a dense stromal microenvironment and is sustained by a cancer-associated fibroblast (CAF)-rich microenvironment, in which reciprocal Wnt/β-catenin signaling between stromal cells, tumor cells, and OCSCs is amplified by CAF-secreted Wnt ligands and other pro-tumorigenic factors that collectively drive ECM remodeling, TCF-mediated transcription, stemness, EMT, and metastatic progression. Strategies targeting this axis, including SFRP4-derived micropeptides, hold promise for disrupting tumor–stromal crosstalk, thereby enhancing sensitivity to platinum- and taxane-based chemotherapy and reducing disease recurrence. Our previous findings demonstrate that SFRP4-derived micropeptides, SC301 and SC401, corresponding to the netrin-like domain (NLD) and cysteine-rich domain (CRD), respectively, sensitize OCSCs to cisplatin by suppressing aberrant Wnt/β-catenin signaling in ovarian cancer cell lines [34,153]. Building on these findings, we propose a future strategy in which SC301/SC401 micropeptides could be conjugated with engineered nanoparticles to facilitate targeted delivery and enhance chemo-sensitization by suppressing the Wnt pathway. This strategy can be further investigated in CSC-enriched populations derived directly from patient material to assess therapeutic responses and chemoresistance. Nanoparticles embedded within hydrogels possess a highly hydrated 3D polymeric network that could protect encapsulated micropeptides from premature degradation by enabling controlled release in response to TME cues. Furthermore, these nanoparticles would also reach the tumor site and stromal compartment, which would enable longer exposure of the micropeptide to CAFs and OCSCs. The controlled release of SC301 or SC401 would thus inhibit Wnt/β-catenin signaling and CAF-mediated resistance and restore tumor sensitivity to chemotherapy. Hydrogel nanoparticle-mediated delivery of SC301 or SC401 would eventually achieve localized stromal reprogramming, which would in turn minimize systemic peptide loss and off-target effects. These proposed effects require experimental validation in tumor models. For clinical translation, pre-treatment biopsy- or liquid biopsy-derived 3D culture platforms could provide predictive biomarkers to identify patients most likely to benefit from Wnt-targeted therapies.

## 12. Conclusions

The lethality of ovarian cancer reflects the failure of cytotoxic chemotherapy to eradicate ovarian cancer stem cells that maintain tumorigenesis, remodel the TME, exploit quiescence, increase drug efflux activity, and increase DNA damage tolerance. Specific markers of CSCs have been identified in a combinatorial manner within the tumor bulk, which reflects their biological identity. PDOs provide physiologically relevant 3D models that preserve aspects of tumor heterogeneity while enabling the study of tumor–TME interactions that 2D cultures fail to capture. However, CSC-derived organoids generated by enriching CSCs from OC patient samples may not recapitulate stromal, immunological, or vascular components unless these factors are included in the culture system. This modular approach enables a controlled investigation of OCSC-specific TME interactions rather than the simultaneous effects of multiple cell types. These models also minimize reliance on animal models. An emerging framework for micropeptide-based treatment, whether derived from short open reading frames or engineered from tumor-suppressor functional domains, offers a conceptual and practical approach to restoring dysregulated signaling pathways. Evidence reviewed here suggests that these molecules can engage with specificity at the level of protein–protein interactions that interrupt oncogenic networks. Computational mapping of functional suppressor domains, together with peptide engineering to enhance stability, bioavailability, and nanocarrier delivery, has advanced the development of synthetic micropeptides. Integrating all the elements in clinical models offers an interdisciplinary path for collaboration across cancer biology, structural bioinformatics, chemical biology, and nanomedicine. Organoid-guided micropeptide therapeutics will facilitate the development of signaling pathway-driven targeted therapies for ovarian cancer stem cells.

## Figures and Tables

**Table 1:** Culture conditions influencing the expansion, maintenance, and translational fidelity of organoid models.

#	Culture Condition	Effect on Organoid Expansion and Fidelity	Organoid/PDO Model Studied
1	Serum-free formulations	Support robust expansion, maintain genetic and phenotypic fidelity, and simplify workflows	Patient-derived tumoroids from colorectal, lung, pancreas, and head and neck cancers [60]; patient-derived tumoroids from colorectal, lung, head and neck, breast, pancreas, and endometrial cancers [61]; general organoid culture systems [62]
2	Conditioned medium and undefined factors	Promotes growth and expansion, but variability and undefined components can affect reproducibility	General organoid culture systems [62] and PDOs from gastric, colorectal, and cervical cancers [63]
3	Defined growth factors	Essential for some organoid types; specific factors can enhance or inhibit growth depending on tumor type	Murine intestinal and mammary organoids; human colon organoids [64]; multiple cancer organoid models [65]
4	Media complexity (permissive vs. minimal)	More complex media yield higher success and long-term expansion, and avoid overgrowth	Murine intestinal and mammary organoids; human colon organoids [64]
5	Media optimization for clinical applications	Media composition alters genotypic and phenotypic fidelity, impacting clinical relevance. Optimized media composition improves the accuracy and utility of CSC-derived organoids	PDOs from gastrointestinal cancers, including pancreatic ductal adenocarcinoma, cholangiocarcinoma, and colorectal adenocarcinoma [66]

**Table 2:** Endogenous peptides encoded by lncRNAs and circRNAs with oncogenic and tumor-suppressive functions.

Peptides	Length (Amino Acids)	Origin	Molecular Mechanism	Primary Cancer Function	Reference
ASAP	94	lncRNA-*LINC00467*	Overexpression modulates the ATP synthase activity and mitochondrial oxygen consumption	Oncogenic	[87]
SMIM30	59	lncRNA-*LINC00998*	Binds the non-receptor tyrosine kinases SRC/YES1 to facilitate their membrane anchoring and phosphorylation, thereby triggering the MAPK signaling pathway.	Oncogenic	[88]
HOXB-AS3’s peptide	53	lncRNA- *HOXB-AS3*	suppressing the PKM splicing that reprograms metabolic demands	Tumor suppressive	[89]
SPAR	90	lncRNA- *LINC00961*	works by acting as a glutamine inhibitor and stabilizing the P27KIP1 protein, and triggering the translocation of cytoplasmic P27KIP1 to lysosomes, where it competitively binds to LAMTOR1, hence suppressing mTORC1 signaling	Tumor suppressive	[90,91]
PINT87aa	87	CircRNA- circular form of *LINC-PINT* exon 2	Direct association with the polymerase-associated factor complex (PAF1c) and suppresses the transcriptional elongation of several oncogenes	Tumor suppressive	[92]
MIAC	51	lncRNA-*AC025154.2*	MIAC directly binds to the AQP2 protein, lowers EREG/EGFR expression, and promotes the downstream pathways PI3K/AKT and MAPK to elicit anti-tumor actions	Tumor suppressive	[93]
APPLE	90	lncRNA-*ASH1L-AS1*	Appears as a novel translation machinery component that stimulates the essential PABPC1-eIF4G interaction, which increases translation initiation and leads to malignancy.	Oncogenic	[94]
AF127577.4-ORF	29	lncRNA- *AF127577.4*	inhibits cell proliferation by reducing the stability of m6A writer methyltransferase like 3 (METTL3), a pro-tumorigenic factor which increases RNA m6A methylation through alteration of the ERK2/METTL3 association.	Tumor suppressive	[95]
CircPPP1R12A-73aa	73	Circular RNA from *PPP1R12A*; peptide translated from an internal ORF	Upregulates the Hippo-YAP signaling pathway to increase proliferation and metastasis.	Oncogenic	[96]

**Table 3:** Micropeptides derived from tumor suppressor protein domains and their mode of action.

Micro Peptide	Source/Domain	Peptide Length	Mechanism of Anti-Tumor Action	Key Outcome/Effect	Reference
SC301, SC401	SFRP 4 (CRD & NLD domains)	17, 20	Blocks Wnt ligand–FZD interaction; disrupts β-catenin stabilization; inhibits β-catenin–CD24 interaction; reduces CSC traits	Reduces stemness, induces apoptosis, and inhibits tumor growth in ovarian cancer cell lines.	[34,101]
PNC21, PNC27, PNC28	mdm-2 binding domain of p53	26, 32, 27	Independent of p53-induced apoptosis	Promotes cell death only in abnormally proliferating cells, observed in lung, cervical, colon, breast, and osteosarcoma human cancer lines.	[100]
P44	SMAR1 (33-mer peptide within the 160 –350-aa region conforming to its RS domain conjugated with 11-mer PTD of TAT protein)	44	Arrests the cell cycle at G2/M phase by p53 accumulation in the nucleus, which is a known property of wild-type p53, and shows a p53-dependent p21-growth suppression mechanism.	Reduced expression of genes affecting mitosis, MAPK signaling, and cell cycle checkpoints, highlighting its role as a cell cycle regulator when treated in CRC and breast carcinoma cell lines.	[102]
PS5	SOCS1-KIR region (52–67 region)	16	Inhibits JAK/STAT signaling by reducing STAT1-STAT3 phosphorylation and suppresses proliferation and survival.	JAK/STAT pathway modulation shown in vascular smooth muscle cells results in anti-inflammatory activity and NK-cell activation, and anticancer activity observed in breast cancer cell lines.	[103,104,105]
IF7	Carbohydrate mimetic peptides (CMP)	7	Selectively binds Annexin A1 (ANXA1) expressed on tumor endothelial cells for rapid vascular targeting and receptor-mediated transcytosis.	Enhances tumor-selective drug delivery, reducing systemic toxicity and increasing apoptosis in Lewis lung carcinoma tumor xenografts.	[106]
ABT-510, ABT-526	2nd type-1 repeat in the stalk region of TSP-1	9	Mimics the anti-angiogenic activity of TSP-1 by binding to endothelial cell receptors.	Inhibits angiogenesis and suppresses tumor neo-vascularization. Progressed to Phase II clinical evaluation.	[107]
ARTS-derived AIBM peptide (Pep3)	C-terminus of Mitochondrial ARTS -IBM (AIBM) protein domain of the tumor suppressor protein ARTS; derived from the unique 27-aa C-terminal region	9	Binds the BIR3 domain of XIAP, promotes XIAP degradation, relieves caspase inhibition, activates caspase-9 and caspase-3, and induces intrinsic apoptosis selectively in cancer cells.	Induces selective apoptosis of cancer cells, reduces XIAP levels, activates caspase-9/-3, demonstrates minimal toxicity to normal cells in a chronic myelogenous leukemia cell line, and provides proof-of-concept for ARTS-based peptide therapeutics.	[108,109]

**Table 4:** Engineering strategies to address limitations of micropeptides and corresponding optimization strategies.

Requirement	Challenge	Stabilization Strategy	Outcome	Reference
Molecular Stability	Rapid proteolytic degradation of micropeptides, which limits in vivo half-life and bioavailability	D-amino acid substitution, Cyclization, Terminal modifications	Increased serum stability, slower clearance, extended therapeutic action	[117,118]
Cell Penetration	Most endogenous functional motifs are not cell-permeable and prevent intracellular delivery to targets	Cyclization, Lipidation, CPP conjugation,	Enhanced membrane crossing, cytosolic/nuclear localization	[119,120,121]
Specificity of Target Interaction	Potential off-target binding or weak affinity of short motifs may reduce efficacy or cause unintended signaling effects	Computational docking & molecular dynamics refinement, Cyclization, Peptidomimetics	Improved binding kinetics, high target selectivity	[122,123]
Pharmacokinetics & Biodistribution	Fast renal clearance due to small molecular size reduces tumor accumulation and systemic exposure	Nanocarrier encapsulation, Lipidation	Extended circulation time, improved EPR-based tumor uptake	[120,124]
Functional Mimicry of Endogenous Domains	Loss of structural context compared to the full-length protein and its natural activity may require minimal structural conformation	Cyclization, Peptidomimetics	Rescues the biologically active conformation	[125,126]
Delivery Challenges	Degradation in serum and poor tissue penetration affect therapeutic delivery to tumors or CSC-rich niches	Nanocarrier encapsulation, CPP conjugation	Improved systemic durability and targeted delivery	[120,121]
Manufacturing & Scalability	Costly synthesis of modified micropeptides impacts feasibility for translational development	Shortened minimal active sequences and Modular peptide production	Cost-effective synthesis and reproducibility	[123]
Immunogenicity	Potential immune recognition of peptide epitopes, which may limit repeated dosing	Nanocarrier encapsulation	Lower immunogenic risk	[120,124]
Functional Potency	Micropeptides may have weak intrinsic activity and should reach threshold activity for tumor suppression	Terminal modifications, Lipidation, Nanocarrier encapsulation, and Co-delivery with synergistic agents	Increased potency and signaling inhibition	[118,120,127]
TME Constraints	Hypoxia, proteases, and acidic pH degrade micropeptides, reducing efficacy in-vivo.	Nanocarrier encapsulation, Terminal modifications, Lipidation, D-amino acid substitution,	Maintained activity within TME	[119,120,128,129]

## Data Availability

No new data were created or analyzed in this study. Data sharing is not applicable to this article.
